# Comparative analysis of four nutritional scores in predicting adverse outcomes in biopsy-confirmed diabetic kidney Disease

**DOI:** 10.3389/fnut.2024.1352030

**Published:** 2024-03-20

**Authors:** Lingzhi Xing, Jiachuan Xiong, Qiyuan Hu, Wenqing Li, Ling Chen

**Affiliations:** ^1^Faculty of Pediatrics, Chongqing Medical University, Chongqing, China; ^2^The Center of Experimental Teaching Management, Chongqing Medical University, Chongqing, China; ^3^Department of Nephrology, The Key Laboratory for the Prevention and Treatment of Chronic Kidney Disease of Chongqing, Chongqing Clinical Research Center of Kidney and Urology Diseases, Xinqiao Hospital, Army Medical University (Third Military Medical University), Chongqing, China

**Keywords:** prognostic nutritional index, geriatric nutritional risk index, triglycerides × total cholesterol × body weight index, controlling nutritional status, end-stage kidney disease, cardiovascular disease, all-cause mortality, renal histologic changes

## Abstract

Malnutrition is associated with adverse outcomes in patients with diabetic kidney disease (DKD). However, it is uncertain which nutritional assessment tools are most effective in predicting the adverse outcomes of DKD. This retrospective study was conducted at a single center and included 367 patients diagnosed with DKD based on biopsy results between August 2009 and December 2018. Four nutritional assessment indices, namely the Prognostic Nutritional Index (PNI), Geriatric Nutritional Risk Index (GNRI), Triglycerides (TG) × Total Cholesterol (TC) × Body Weight (BW) Index (TCBI), and Controlling Nutritional Status (CONUT) score, were selected and calculated. We aimed to assess the association between these nutritional scores and adverse outcomes, including progression to end-stage kidney disease (ESKD), cardiovascular diseases events (CVD), and all-cause mortality. Univariate and multivariate Cox regression analyses, Kaplan–Meier analysis, along with Restricted cubic spline analysis were used to examine the relationship between nutritional scores and adverse outcomes. Furthermore, the area under the curve (AUC) was calculated using time-dependent receiver operating characteristics to determine the predictive value of the four nutritional scores alone and some combinations. Lastly, ordered logistic regression analysis was conducted to explore the correlation between the four nutritional scores and different renal histologic changes. The incidence of ESKD, CVD, and all-cause mortality was significantly higher in patients with DKD who had a lower PNI, lower GNRI, and higher CONUT score. Additionally, The TCBI performed the worst in terms of grading and risk assessment. The PNI offer the highest predictive value for adverse outcomes and a stronger correlation with renal histologic changes compared to other nutritional scores. Patients diagnosed with DKD who have a worse nutritional status are more likely to experience higher rates of adverse outcomes. The PNI might offer more valuable predictive values and a stronger correlation with different renal histologic changes compared to other nutritional scores.

## 1 Introduction

Diabetic kidney disease (DKD) has become a major public health concern, with a high incidence rate, high mortality, and high medical costs (1, 2). Nutritional status is closely related to the progression of end-stage kidney disease (ESKD), cardiovascular events (CVD), and all-cause mortality (3, 4). Therefore, evaluating the nutritional status of DKD patients and using it to assess the occurrence, development, and prognosis of DKD is extremely important. Currently, four objective nutritional scores have been used in previous studies to evaluate the prognosis of patients with DKD. These scores include the Prognostic Nutrition Index (PNI) (5), Geriatric Nutritional Risk Index (GNRI) (6), Triglycerides (TG) × Total cholesterol (TC) × Body weight (BW) index (TCBI) (7), and controlling nutritional status (CONUT) score (8). Previous studies have found that GNRI and PNI are effective tools in assessing the prognosis of patients with chronic kidney disease (CKD) (9, 10). CONUT score has also been identified as an independent risk factor for ESKD, CVD events, and overall death in patients with DKD (11). According to a recent study comparing GNRI, PNI, and TCBI, PNI has the most significant predictive value for all-cause and cardiovascular mortality in the general population (12)^.^ Additionally, previous studies have shown that renal histological changes are good predictors of ESKD (13). However, the relationship between these four nutritional scores and adverse outcomes remains elusive in patients with DKD. It is also still unclear which score or combination is more valuable in predicting the adverse outcomes. Additionally, the correlation between nutritional status and renal histologic changes in patients with DKD is largely unknown. Therefore, this study aims to introduce four nutritional scores to evaluate the nutritional status of DKD patients with different renal histologic changes. It also aims to analyze in-depth the correlation between nutritional status and ESKD, CVD events and all-cause death, and the correlation between nutritional status and different renal histologic changes in DKD patients. The ultimate goal is to provide new ideas for the prevention and treatment measures of disease occurrence, development, and prognosis in clinical DKD patients.

## 2 Materials and methods

### 2.1 Data source and case selection

**Figure 1:**
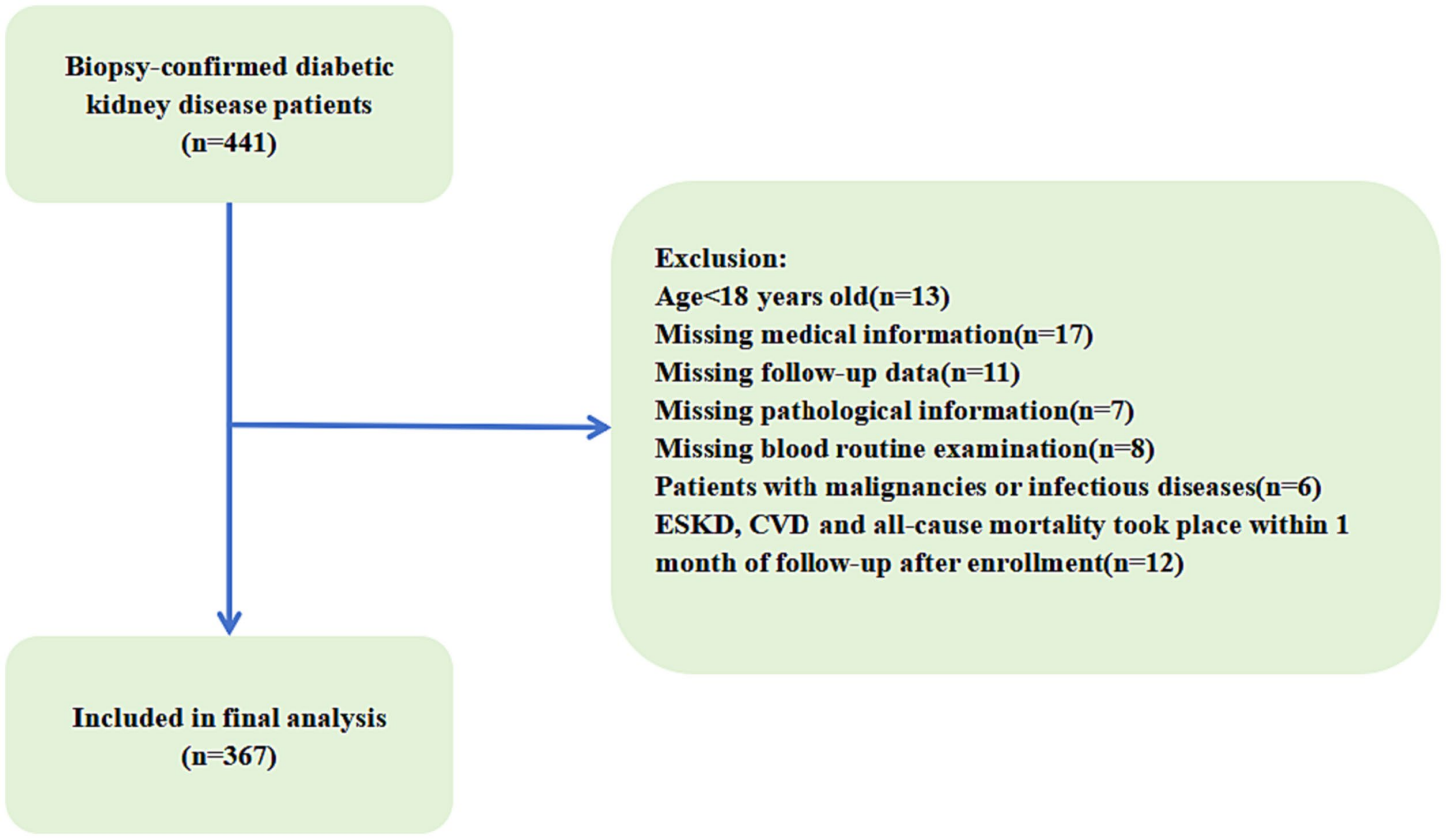
Flowchart of included patients in this study. ESKD, end-stage kidney disease; CVD, cardiovascular disease.

This retrospective study included 367 patients with biopsy-confirmed DKD from Xinqiao Hospital of the Army Medical University in China between August 2009 and December 2018. DKD was diagnosed based on criteria established by the Renal Pathology Society in 2010 (14). All participants were followed up from the screening date until 31 December 2021 or until their death. The study protocol was approved by the ethical committee of Xinqiao Hospital (No. 2018-006-02). Inclusion criteria were: (1) biopsy-confirmed DKD; (2) adults aged 18 years or older; (3) complete medical information and follow-up data. Exclusion criteria were: (1) end-stage kidney disease (ESKD), cardiovascular (CVD) events and all-cause death took place within 1 month of follow-up after enrollment; (2) patients with incomplete pathological information or blood routine examination; (3) patients with malignancies (e.g., breast, lung, gastrointestinal, hematologic cancers), infectious diseases (e.g., pneumonia, viral hepatitis) (Figure 1).

### 2.2 Clinical information acquisition

We extracted baseline demographic characteristics and laboratory values from the Electronic Medical Record System of Xinqiao Hospital at the time of the patient’s first renal biopsy. This included demographic data such as age and gender, medical history including hypertension and history of coronary heart disease, and laboratory data such as lymphocyte count, hemoglobin, serum creatinine, blood urea nitrogen (BUN), uric acid, intact parathyroid hormone (iPTH), calcium, magnesium, phosphate, albumin, total cholesterol, low-density lipoprotein (LDL) cholesterol, high-density lipoprotein (HDL) cholesterol, triglycerides (TG), proteinuria, and pathological information. We determined the estimated glomerular filtration rate (eGFR) using the cystatin C-based chronic kidney disease (CKD)-EPI equation and combined by serum creatinine (CKD-EPIscr-cys) which incorporates the Chinese eGFR racial factor.

### 2.3 Preliminary data processing

We calculated body mass index (BMI) by dividing weight in kilograms by height in meters squared based on the obtained height and weight measurements. In addition, the PNI was defined by the following formula: PNI = serum albumin (g/L) + 5 × total lymphocyte count (10^9^/L). The GNRI was calculated by using the following formula: GNRI = [1.489 × serum albumin (g/l)] + [41.7 × weight (kg)/ideal body weight (kg)]. The calculation of the ideal body was as follows: 22 × square of height because of its validity. The ratio of weight-to-ideal body weight was set to 1 if the actual body weight exceeded the ideal body weight (15). The TCBI was calculated using the formula: serum level of TG (mg/dL) × TC (mg/dL) × body weight (kg)/1,000. The CONUT score was described in Supplementary Table S1 (8). Nutritional scores (including GNRI, PNI, TCBI, CONUT) were divided into four groups according to the mean four nutritional scores of the quartiles: Q1 (GNRI <82.35), Q2(GNRI:82.35–92.92), Q3 (GNRI:92.92–102.90) and Q4 (GNRI >102.90) GNRI groups; Q1 (PNI < 35.26), Q2 (PNI: 35.26–42.75), Q3 (PNI:42.75–50.19) and Q4(PNI > 50.19) PNI groups; and Q1 (TCBI <1,210.17), Q2 (TCBI: 1,210.17–2079.83), Q3 (TCBI:2079.83–3451.43) and Q4(TCBI >3451.43) TCBI groups. For the CONUT score, a score of 0 was considered Q1, scores of 1 to 2 were considered Q2, scores of 3 to 4 were considered Q,2 and scores of≥5 were considered Q4 CONUT groups.

### 2.4 Clinical outcomes

The study evaluated three outcomes: ESKD, CVD events, and all-cause mortality, each of which was considered separately. ESKD was defined as an eGFR less than 15 mL/min/1.73 m^2^ or the need for maintenance renal replacement therapy due to irreversible deterioration of renal function, including hemodialysis, peritoneal dialysis, or kidney transplantation. CVD events were defined as the occurrence of new CVD events, such as coronary heart disease, heart failure, cerebrovascular events, and severe arrhythmia. All-cause mortality was defined as death from any cause. The study obtained clinical outcomes primarily through telephone follow-up or patient medical record reports.

### 2.5 Statistical analysis

The data analysis involved the use of SPSS (version 27.0), GraphPad Prism (version 10.0.3) or R version 4.3.1. All the data used were checked for normality of distribution using the Kolmogorov–Smirnov test. Normally distributed data were expressed as mean ± standard deviation while non-normally distributed data were expressed as median (interquartile range). The differences between groups were tested using *t*-tests, Mann–Whitney U tests, and chi-square tests. The Kaplan–Meier curve was used to compare the outcomes of the patients according to the mean four nutritional scores (including PNI, GNRI, TCBI, and CONUT) of the quartiles, and the log-rank test was used to compare the differences between each group. Furthermore, the independent relationships between four nutritional scores and end-stage renal disease, cardiovascular, and all-cause mortality were investigated by univariate and multivariate Cox regression models. The initial confounding factors were selected based on previous studies, data availability, and established associations. If these factors changed the estimates of four nutritional scores on end-stage renal disease, cardiovascular, and all-cause mortality by more than 10% or were significantly associated with endpoint events after adjustment for sociodemographic factors (age), they were included as the covariates in multivariate Cox regression analysis. Hazard ratios (HRs) and 95% confidence intervals (CIs) are provided. Additionally, restricted cubic splines (RCS) and threshold effect analysis were applied using the R package “rms” based on the Cox proportional hazards models to further explore the relationship between the nutritional scores and endpoint events. Moreover, the time-dependent receiver operating characteristic (td-ROC) curve was constructed to compare the diagnostic accuracy of PNI, GNRI, TCBI, and CONUT, alone or in different combinations with PNI, in predicting end-stage renal disease, cardiovascular and all-cause mortality. Combine four nutritional scores in the following different ways: PNI + CONUT, PNI + TCBI, PNI + GNRI, PNI + TCBI+CONUT, PNI + GNRI+CONUT, PNI + GNRI+TCBI, and PNI + TCBI+CONUT+GNRI. Then, the area under the curve (AUC) was calculated for different groups. The correlation heatmap was generated using R software (v.4.2.2) package “corrplot” (v.0.92) (16) and “ggplot2” (v3.4.2) (17). To explore the correlation between four nutritional scores and different renal histologic changes (glomerular lesions, IFTA, interstitial inflammation, arteriolar hyalinosis, and arteriosclerosis) in patients with diabetic kidney disease, we used X-tile software (v3.6.1) to calculate the optimum cutoff value for converting the continuous variables (PNI, GNRI) into categorical variables (the low-level group and the high-level group) according to end-stage renal disease. Patients were divided into two groups based on median CONUT score. Then, we use ordered logistic regression analysis to investigate the factors affecting different renal histologic changes in patients with diabetic kidney disease. Ordered logistic regression analysis: The model meets the parallelism by parallel test, and multivariate analysis was performed by ordinal logistic regression. It is important to note that a *p* value of <0.05 was considered statistically significant during the analysis.

## 3 Results

### 3.1 Baseline characteristics

**Figure 2:**
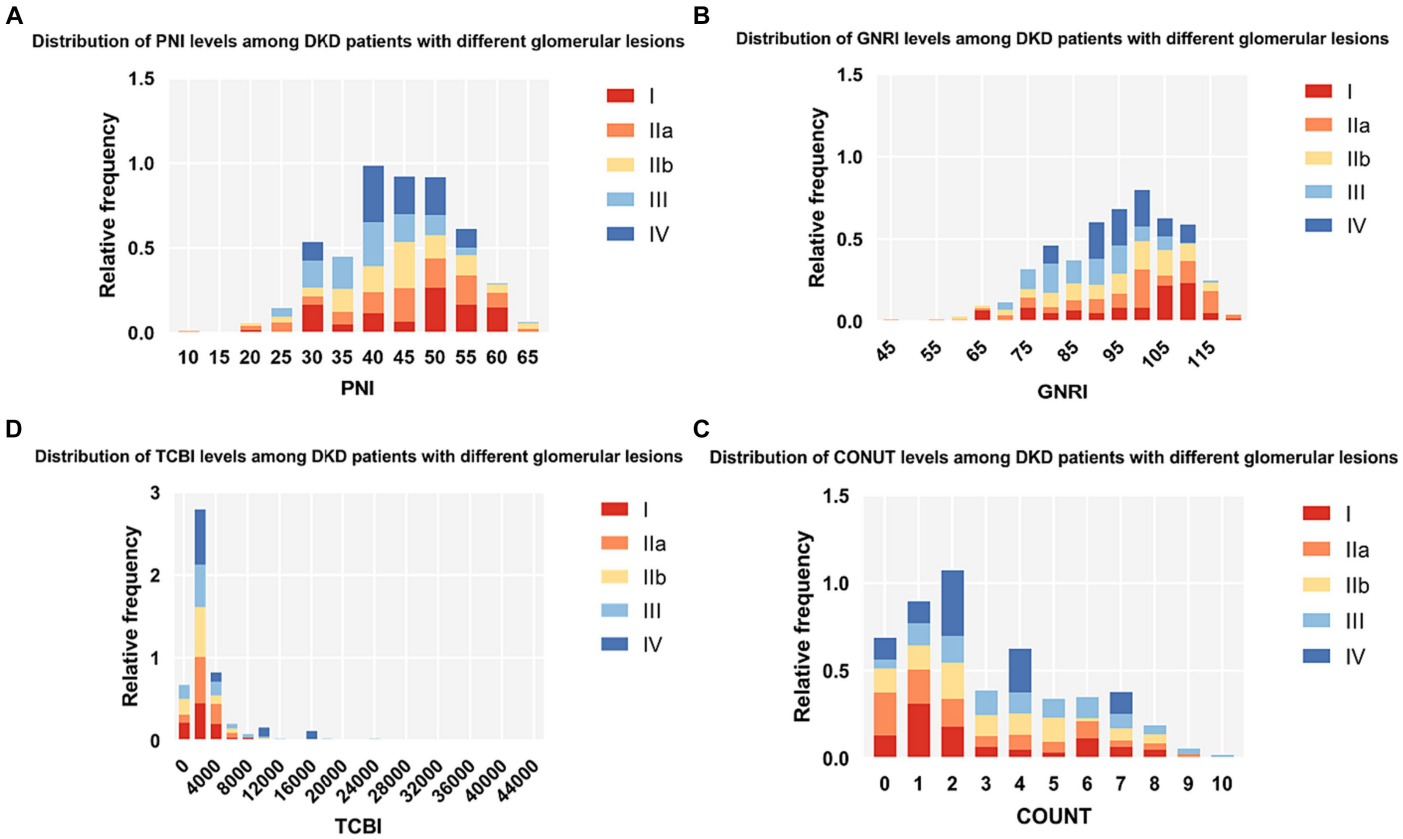
Histograms show the population distribution of nutritional scores. **(A)** PNI; **(B)** GNRI; **(C)** TCBI; **(D)** COUNT score. PNI, Prognostic Nutritional Index; GNRI, Geriatric Nutritional Risk Index; CONUT score, Controlling Nutritional Status score; TCBI, Triglycerides×Total Cholesterol×Body Weight Index.

A total of 367 patients with DKD were recruited for the current study. The mean age of the patients was 51.30 ± 10.35 years, and 63.2% (232) were male. Among the patients, 34.1% (125) were smokers, 70.8% (260) had hypertension, and 20.7% (76) had a history of coronary heart disease. Then, the PNI, GNRI, TCBI, and CONUT score were calculated. The baseline characteristics of the study population according to different glomerular lesions are shown in Table 1. According to our findings, serum creatinine, uric acid, blood urea nitrogen, Cystatin C, LPA, TNF-α, and CONUT increased in proportion to the severity of glomerular lesions, and patients with more severe glomerular lesions were more likely to suffer from coronary heart disease and diabetes retinopathy. They also have lower levels of hemoglobin, eGFR, albumin, GNRI, and PNI. In addition, we found that significant differences in interstitial fibrosis and tubular atrophy (IFTA), interstitial inflammation, arteriolar hyalinosis, and arteriosclerosis. The distribution of the PNI, GNRI, TCBI, and CONUT score among DKD patients with different glomerular lesions is shown in Figure 2.

**Table 1:** Baseline characteristics of patients with diabetic kidney disease.

valuable	ALL (*n* = 367)	Class I (*n* = 60)	Class II a (*n* = 81)	Class II b (*n* = 60)	Class III (*n* = 158)	Class IV (*n* = 8)	*p* value
Gender (Male, %)	232 (63.2%)	35 (58.3%)	49 (60.5%)	36 (60.0%)	109 (69.0%)	3 (37.5%)	0.224
Age (years)	51.30 ± 10.35	50.55 ± 11.76	53.44 ± 10.13	49.55 ± 10.21	51.20 ± 10.01	50.5 ± 7.31	0.234
Smoking (%)	125 (34.1%)	18 (30.0%)	28 (34.6%)	24 (40.0%)	54 (34.2%)	1 (12.5%)	0.550
Hypertension (%)	260 (70.8%)	27 (45.0%)	50 (61.7%)	41 (68.3%)	135 (85.4%)	7 (87.5%)	<0.001
Coronary disease (%)	76 (20.7%)	7 (11.7%)	18 (22.2%)	9 (15.0%)	39 (24.7%)	3 (37.5%)	0.126
Diabetic retinopathy (%)	211 (57.5%)	17 (28.3%)	34 (42.0%)	41 (68.3%)	114 (72.2%)	5 (62.5%)	<0.001
Neutrophil (%)	65.40 (59.40–71.60)	64.80 (58.13–69.80)	64.40 (58.30–68.60)	66.85 (60.43–71.75)	66.40 (61.38–72.48)	71.35 (64.98–76.83)	0.040
Medication							
Statin use (%)	75 (20.4%)	8 (13.3%)	11 (13.6%)	11 (18.3%)	43 (27.2%)	2 (25.0%)	0.063
Anti-platelet drug use (%)	51 (13.9%)	7 (11.7%)	7 (8.6%)	5 (8.3%)	31 (19.6%)	1 (12.5%)	0.091
Laboratory data							
Lymphocyte (%)	24.30 (18.60–29.10)	24.40 (21.93–32.98)	25.10 (21.00–31.65)	22.95 (18.15–26.65)	23.90 (17.80–27.63)	19.75 (16.45–24.20)	0.025
Proteinuria (g/day)	2.48 (0.74–5.28)	0.81 (0.23–3.98)	0.96 (0.29–2.98)	1.89 (0.91–4.13)	4.32 (2.17–7.20)	2.63 (1.34–4.86)	<0.001
Fasting glucose (mmol/L)	6.82 (5.30–9.10)	7.00 (5.78–8.62)	6.29 (4.95–8.94)	7.04 (5.00–9.15)	6.89 (5.30–9.49)	5.02 (3.53–7.96)	<0.001
HbA1c (%)	7.30 (6.50–8.80)	7.55 (6.43–8.58)	7.10 (6.50–8.25)	7.20 (6.50–8.45)	7.55 (6.50–9.34)	7.10 (6.34–9.28)	<0.001
SBP (mmHg)	144.40 ± 22.96	132.02 ± 17.02	138.21 ± 22.88	142.67 ± 18.14	152.78 ± 23.54	147.63 ± 23.56	<0.001
DBP (mmHg)	84.00 (77.00–93.00)	82.00 (73.00–89.00)	82.00 (75.00–90.00)	84.00 (78.25–90.00)	86.00 (78.00–96.00)	90.00 (80.50–95.00)	0.044
Height (cm)	163.31 ± 7.78	162.84 ± 8.29	163.96 ± 7.73	161.98 ± 7.76	163.92 ± 7.49	158.31 ± 8.64	0.145
Weight (kg)	67.19 ± 11.49	68.23 ± 11.72	68.80 ± 11.64	64.48 ± 11.67	67.18 ± 11.13	63.55 ± 12.52	0.183
BMI (kg/m^2^)	25.10 ± 3.30	25.61 ± 3.04	25.52 ± 3.55	24.45 ± 3.13	24.92 ± 3.23	25.32 ± 4.44	0.229
Hemoglobin (g/L)	116.24 ± 27.20	130.97 ± 19.31	127.86 ± 24.05	116.17 ± 35.26	104.98 ± 22.67	110.88 ± 24.67	<0.001
eGFR (ml/min/1.73 m^2^)	64.00 (37.00–97.85)	95.97 (71.58–112.59)	90.92 (62.84–110.67)	66.15 (36.52–102.56)	43.04 (27.48–64.27)	32.50 (24.74–73.62)	<0.001
Serum creatinine (μmol/L)	110.00 (71.40–155.20)	72.00 (57.18–94.78)	77.50 (63.25–101.95)	109.75 (71.05–151.55)	144.25 (111.08–216.45)	185.25 (92.13–203.48)	<0.001
Uric acid (μmol/L)	384.70 ± 105.81	347.85 ± 112.07	371.78 ± 112.12	398.79 ± 116.17	398.09 ± 93.25	421.63 ± 79.01	0.010
BUN (mmol/L)	7.33 (5.52–9.99)	5.53 (4.80–7.63)	6.28 (5.07–7.94)	7.43 (5.38–9.88)	8.66 (6.58–10.86)	8.41 (6.72–11.68)	<0.001
Cystatin C (mg/L)	1.49 (1.03–2.20)	1.00 (0.75–1.38)	1.08 (0.88–1.54)	1.39 (1.04–2.17)	1.92 (1.45–2.66)	1.90 (1.52–2.90)	<0.001
Calcium (mmol/L)	2.18 (2.05–2.30)	2.20 (2.09–2.34)	2.25 (2.10–2.36)	2.21 (2.08–2.32)	2.15 (2.03–2.24)	2.13 (1.96–2.34)	<0.001
Phosphorus (mmol/L)	1.16 (1.03–1.34)	1.13 (1.02–1.29)	1.06 (0.92–1.24)	1.15 (1.03–1.34)	1.23 (1.09–1.40)	1.19 (0.99–1.36)	<0.001
iPTH (pg/mL)	64.30 (39.60–92.30)	56.30 (39.00–64.30)	61.20 (37.10–77.50)	53.15 (36.04–72.10)	67.45 (41.80–125.73)	93.70 (53.28–153.43)	0.002
Albumin (g/L)	34.53 ± 8.86	37.30 ± 9.74	37.27 ± 10.17	35.38 ± 8.59	31.62 ± 7.03	37.00 ± 6.41	<0.001
Lpa (mg/L)	275.00 (113.00–597.0)	131.00 (40.75–300.75)	248.00 (83.00–458.00)	264.00 (124.25–524.00)	387.00 (169.00–781.75)	394.00 (157.00–1006.00)	<0.001
APOA1 (g/L)	1.32 (1.12–1.56)	1.27 (1.09–1.58)	1.28 (1.09–1.49)	1.32 (1.11–1.55)	1.37 (1.14–1.58)	1.31 (1.08–1.75)	<0.001
APOB (g/L)	1.02 (0.82–1.25)	0.95 (0.74–1.12)	1.04 (0.87–1.24)	1.01 (0.82–1.31)	1.09 (0.84–1.34)	0.87 (0.78–1.15)	<0.001
APOE (mg/dL)	3.86 (3.00–4.37)	3.91 (3.20–5.24)	3.91 (3.06–4.11)	3.74 (2.91–4.04)	3.86 (2.89–4.24)	3.82 (3.04–5.75)	<0.001
Triglycerides (mmol/L)	1.70 (1.19–2.54)	1.82 (1.16–3.12)	1.84 (1.28–2.70)	1.70 (1.15–2.56)	1.64 (1.08–2.36)	1.64 (1.29–6.36)	<0.001
LDL (mmol/L)	3.10 (2.49–4.02)	2.74 (2.31–3.59)	3.18 (2.47–4.00)	2.98 (2.39–3.96)	3.27 (2.61–4.27)	2.53 (2.21–3.03)	0.034
HDL (mmol/L)	1.17 (0.94–1.46)	1.12 (0.91–1.55)	1.07 (0.93–1.34)	1.20 (0.95–1.47)	1.22 (0.97–1.53)	1.00 (0.85–1.87)	<0.001
Total cholesterol (mmol/L)	5.20 (4.19–6.47)	4.88 (4.02–6.01)	5.00 (4.16–6.01)	5.30 (4.25–6.51)	5.51 (4.37–6.98)	5.35 (3.99–6.81)	<0.001
CRP (mg/L)	4.70 (2.50–6.80)	5.00 (2.23–6.80)	5.40 (2.95–6.80)	3.00 (2.05–6.80)	5.10 (2.50–6.80)	3.85 (2.95–6.40)	0.091
TNF-α	10.70 (8.10–11.10)	9.60 (7.00–10.70)	10.40 (8.10–10.80)	10.70 (8.15–11.85)	10.70 (8.48–11.55)	8.65 (7.85–10.68)	0.033
PNI	42.75 (35.26–50.19)	49.49 (37.91–54.50)	46.88 (38.42–54.15)	45.11 (37.54–48.74)	39.81 (33.29–44.16)	43.63 (40.22–47.44)	<0.001
GNRI	92.92 (82.35–102.90)	102.82 (86.93–107.89)	99.62 (87.45–108.74)	95.60 (83.09–104.35)	88.08 (80.67–94.89)	94.56 (91.69–101.13)	<0.001
TCBI	2079.83 (1210.17–3451.43)	2071.94 (1121.05–3907.08)	2281.74 (1414.59–3792.47)	2227.59 (1068.87–2850.11)	1987.10 (1157.46–3448.97)	1681.74 (1189.77–8983.92)	0.791
CONUT score ≥ 3 (%)	195 (53.3%)	23 (38.3%)	33 (40.2%)	30 (51.7%)	106 (67.1%)	3 (37.5%)	<0.001
IFTA							
0	44 (12.0%)	34 (56.7%)	10 (12.20%)	0 (0.0%)	0 (0.0%)	0 (0.0%)	<0.001
1	116 (31.7%)	22 (36.7%)	53 (64.6%)	24 (41.4%)	17 (10.8%)	0 (0.0%)	
2	93 (25.4%)	4 (6.7%)	14 (17.1%)	25 (43.1%)	49 (31%)	1 (12.5%)	
3	113 (30.9%)	0 (0.0%)	5 (6.1%)	9 (15.5%)	92 (58.2%)	7 (87.5%)	
Interstitial inflammation							
0	49 (13.4%)	33 (55.0%)	14 (17.1%)	1 (1.7%)	1 (0.6%)	0 (0.0%)	<0.001
1	115 (31.4%)	15 (25.0%)	52 (63.4%)	24 (41.4%)	24 (15.2%)	0 (0.0%)	
2	202 (55.0%)	12 (20.0%)	16 (19.5%)	33 (56.9%)	133 (84.2%)	8 (100.0%)	
Arteriolar hyalinosis							
0	14 (3.8%)	5 (8.3%)	7 (8.5%)	2 (3.4%)	0 (0.0%)	0 (0.0%)	<0.001
1	92 (25.1%)	37 (61.7%)	27 (32.9%)	16 (27.6%)	10 (6.3%)	2 (25.0%)	
2	260 (71.0%)	18 (30.0%)	48 (58.5%)	40 (69.0%)	148 (93.7%)	6 (75.0%)	
Arteriosclerosis							
0	94 (25.7%)	36 (60.0%)	31 (37.8%)	17 (29.3%)	10 (6.3%)	0 (0.0%)	<0.001
1	141 (38.5%)	17 (28.3%)	33 (40.2%)	24 (41.4%)	64 (40.5%)	3 (37.5%)	
2	131 (35.8%)	7 (11.7%)	18 (22.0%)	17 (29.3%)	84 (53.2%)	5 (62.5%)	

Data are presented as mean ± SD, *N* (%) or median (IQR). HbA1c, glycated hemoglobin; SBP, systolic blood pressure; DBP, diastolic blood pressure; BMI, body mass index; eGFR, estimated glomerular filtration rate; BUN, blood urea nitrogen; iPTH, intact parathyroid hormone; Lpa, Lipoprotein a; LDL, low-density lipoprotein; HDL, high-density lipoprotein; CRP, C-reactive protein; TNF-α, tumor necrosis factor-α; PNI, Prognostic Nutritional Index; GNRI, Geriatric Nutritional Risk Index; TCBI, Triglycerides × Total Cholesterol × Body Weight Index; CONUT score, Controlling Nutritional Status score; IFTA, interstitial fibrosis and tubular atrophy.

### 3.2 Association between four nutritional scores and adverse outcomes

**Figure 3:**
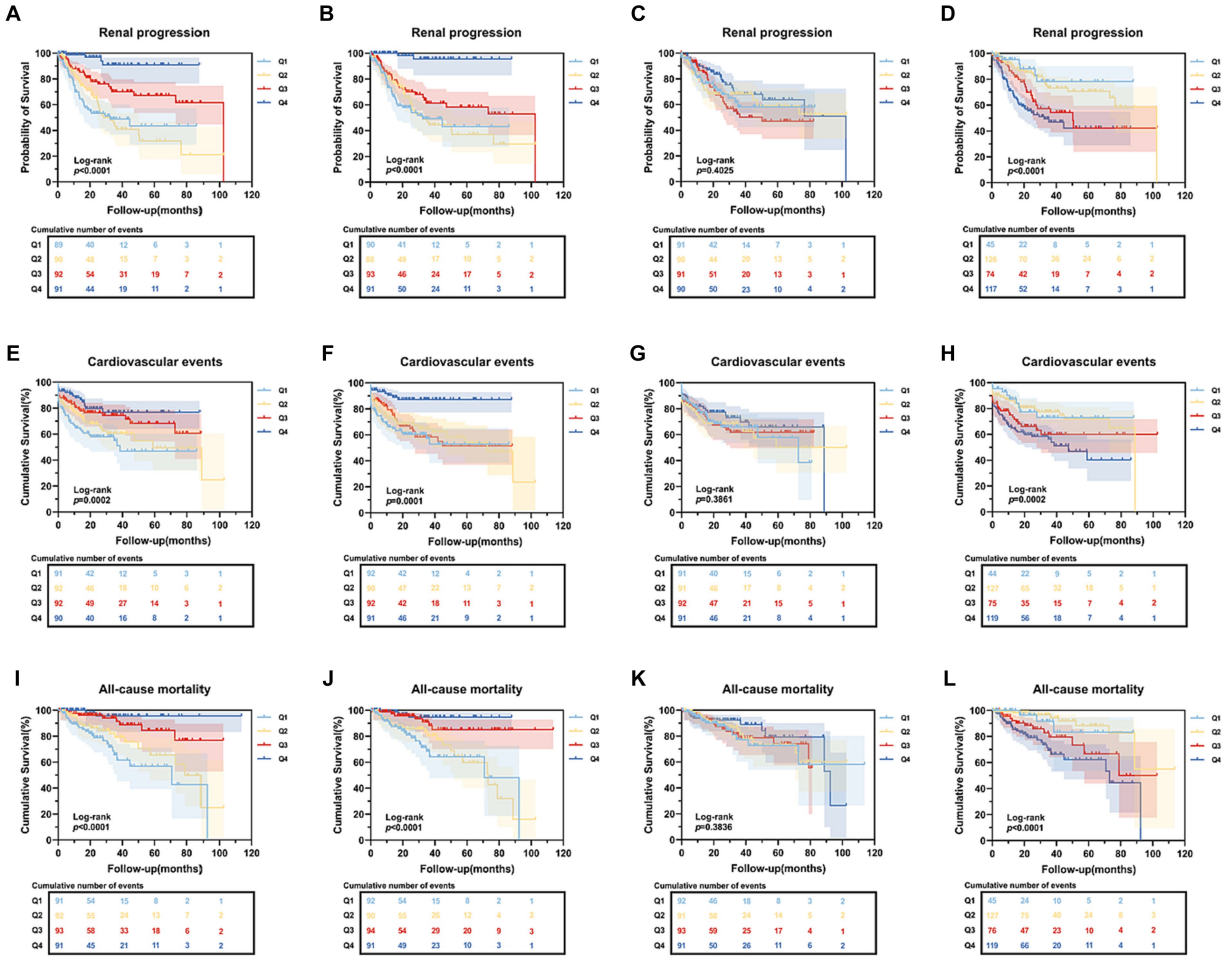
Kaplan–Meier curves of end-stage renal disease, cardiovascular events and all-cause mortality based on four nutritional scores. **(A–D)** Kaplan–Meier curves of end-stage renal disease categorized by PNI, GNRI, TCBI, COUNT score; **(E–H)** Kaplan–Meier curves of cardiovascular death categorized by PNI, GNRI, TCBI, COUNT score; **(I–L)** Kaplan–Meier curves of all-cause mortality categorized by PNI, GNRI, TCBI, COUNT score. PNI, Prognostic Nutritional Index; GNRI, Geriatric Nutritional Risk Index; TCBI, Triglycerides×Total Cholesterol×Body Weight Index; CONUT score, Controlling Nutritional Status score.

During a median follow-up period of 5.1 years, about 114 (31.1%) of ESKD, 115 (31.3%) of CVD events, and 54 (14.7%) of deaths occurred. The Kaplan–Meier curve showed that, PNI, GNRI, and COUNT score were all significantly associated with renal progression, CVD events and all-cause mortality except for TCBI (Figure 3). Then, we performed the Cox regression analysis (Tables 2, 3). In the univariate Cox proportional hazards analysis, patients with lower GNRI, PNI and higher CONUT score had increased risks of ESKD (*p* < 0.001), CVD events (*p* < 0.001) and all-cause mortality (*p* < 0.001). Moreover, age, hypertension, diabetic retinopathy, eGFR, serum creatinine, cystatin C, calcium, hemoglobin, iPTH, albumin, LDL, IFTA, and arteriosclerosis were also significantly associated with adverse outcomes (Table 2). In a multivariate Cox regression model (Model 3), the GNRI and PNI were still associated with the incidence of adverse outcomes. In addition, the CONUT score was still an independent predictor of CVD events (HR = 1.113, 95% CI 1.029–1.203, *p* = 0.007), and all-cause mortality (HR = 1.208, 95% CI 1.033–1.411, *p* = 0.018) in Model 3, but the positive effect size of ESKD was non-significant (Table 3).

**Table 2:** Univariate Cox analysis of adverse outcomes in patients with diabetic kidney disease.

Variable	ESKD		CVD		All-cause mortality	
	HR (95% CI)	*p* value	HR (95% CI)	*p* value	HR (95% CI)	*p* value
Male	0.663 (0.463–0.949)	0.025	0.730 (0.501–1.065)	0.102	1.203 (0.754–1.919)	0.437
Age	1.006 (0.988–1.023)	0.526	1.036 (1.015–1.056)	<0.001	1.037 (1.009–1.066)	0.009
Smoking	1.208 (0.830–1.757)	0.325	1.071 (0.708–1.619)	0.746	1.254 (0.731–2.151)	0.412
Hypertension	2.680 (1.578–4.551)	<0.001	2.156 (1.260–3.688)	0.005	2.754 (1.240–6.119)	0.130
Coronary disease	1.471 (0.973–2.226)	0.067	5.009 (3.341–7.511)	<0.001	1.381 (0.757–2.520)	0.292
Diabetic retinopathy	2.623 (1.692–4.066)	<0.001	1.799 (1.165–2.780)	0.008	2.161 (1.153–4.051)	0.016
CKD stage	1.488 (1.298–1.706)	<0.001	1.326 (1.148–1.531)	<0.001	1.235 (1.028–1.484)	0.024
Statin use	1.646 (1.076–2.518)	0.022	2.504 (1.636–3.833)	<0.001	0.920 (0.445–1.900)	0.822
Anti-platelet drug use	1.342 (0.808–2.230)	0.256	2.262 (1.378–3.712)	0.001	0.991 (0.422–2.327)	0.984
eGFR	0.965 (0.958–0.973)	<0.001	0.985 (0.978–0.991)	<0.001	0.980 (0.972–0.989)	<0.001
Serum creatinine	1.007 (1.006–1.008)	<0.001	1.003 (1.002–1.004)	<0.001	1.004 (1.003–1.006)	<0.001
Uric acid	1.002 (1.000–1.004)	0.012	1.002 (1.001–1.004)	0.110	1.001 (0.999–1.004)	0.294
BUN	1.001 (0.996–1.007)	0.629	1.000 (0.992–1.008)	0.996	1.001 (0.992–1.010)	0.783
Cystatin C	2.429 (2.081–2.837)	<0.001	1.514 (1.267–1.809)	<0.001	1.797 (1.424–2.267)	<0.001
Calcium	0.089 (0.035–0.226)	<0.001	0.269 (0.095–0.760)	0.013	0.88 (0.024–0.320)	<0.001
Phosphorus	1.447 (1.116–1.874)	0.005	1.076 (0.668–1.734)	0.763	1.236 (0.742–2.060)	0.416
Hemoglobin	0.960 (0.951–0.970)	<0.001	0.990 (0.982–0.998)	0.012	0.973 (0.961–0.985)	<0.001
iPTH	1.009 (1.007–1.011)	<0.001	1.006 (1.003–1.008)	<0.001	1.006 (1.002–1.010)	0.007
Albumin	0.942 (0.923–0.962)	<0.001	0.962 (0.941–0.984)	<0.001	0.919 (0.889–0.951)	<0.001
TCH	1.075 (0.976–1.184)	0.141	1.020 (0.910–1.143)	0.732	1.278 (1.114–1.466)	<0.001
TG	0.880 (0.765–1.012)	0.073	0.963 (0.861–1.078)	0.517	0.863 (0.695–1.072)	0.184
Lpa	1.001 (1.000–1.001)	<0.001	1.001 (1.000–1.001)	<0.001	1.001 (1.000–1.001)	0.003
APOA1	0.905 (0.648–1.265)	0.559	0.789 (0.485–1.283)	0.789	1.086 (0.828–1.425)	0.551
APOB	0.943 (0.608–1.463)	0.792	1.081 (0.680–1.719)	0.743	1.456 (0.8116–2.599)	0.203
APOE	0.973 (0.873–1.086)	0.629	0.949 (0.836–1.078)	0.422	1.061 (0.904–1.246)	0.467
HDL-C	1.006 (0.682–1.485)	0.975	0.974 (0.627–1.513)	0.974	1.925 (1.193–3.105)	0.007
LDL-C	1.037 (0.900–1.196)	0.615	0.928 (0.780–1.103)	0.394	1.225 (1.007–1.490)	0.042
CRP	1.000 (0.994–1.005)	0.900	1.001 (0.997–1.006)	0.545	1.001 (0.994–1.008)	0.738
IL-6	1.006 (0.998–1.013)	0.136	1.001 (0,991–1.012)	0.804	1.004 (0.992–1.017)	0.493
TNF-α	1.042 (1.007–1.079)	0.019	1.019 (0.974–1.065)	0.417	1.057 (1.003–1.114)	0.037
IL-8	1.004 (0.995–1.014)	0.378	1.011 (1.003–1.019)	0.004	0.995 (0.965–1.026)	0.763
Glomerular lesions	2.121 (1.719–2.617)	<0.001	1.244 (1.053–1.471)	0.010	1.269 (0.993–1.621)	0.057
IFTA	2.326 (1.860–2.908)	<0.001	1.360 (1.106–1.672)	0.004	1.357 (1.038–1.774)	0.026
Interstitial inflammation	2.540 (1.855–3.476)	<0.001	1.650 (1.212–2.246)	0.001	1.537 (1.023–2.310)	0.390
Arteriolar hyalinosis	2.605 (1.552–4.373)	<0.001	1.286 (0.866–1.910)	0.212	1.811 (0.952–3.447)	0.070
Arteriosclerosis	1.755 (1.361–2.262)	<0.001	1.672 (1.271–2.198)	<0.001	1.764 (1.219–2.552)	0.003

Data are presented as HR (hazard ratios), 95% CI (confidence intervals), and *p* value. CKD, chronic kidney disease; eGFR, estimated glomerular filtration rate; BUN, blood urea nitrogen; iPTH, intact parathyroid hormone; TCH, total cholesterol; TG, triglycerides; Lpa, Lipoprotein a; HDL, High-density lipoprotein; LDL, low-density lipoprotein; CRP, C-reactive protein; TNF-α, tumor necrosis factor-α; IFTA, interstitial fibrosis and tubular atrophy; ESKD, end-stage kidney disease; CVD: cardiovascular.

**Table 3:** Multivariate Cox analysis of adverse outcomes in patients with diabetic kidney disease.

	Model 1		Model 2		Model 3	
	HR (95% CI)	*p* value	HR (95% CI)	*p* value	HR (95% CI)	*p* value
ESKD						
PNI	0.942 (0.924–0.961)	<0.001	0.941 (0.923–0.959)	<0.001	0.963 (0.938–0.989)	0.006
GNRI	0.963 (0.950–0.976)	<0.001	0.961 (0.949–0.974)	<0.001	0.974 (0.956–0.992)	0.004
TCBI	1.000 (1.000–1.000)	0.453	1.000 (1.000–1.000)	0.382	1.000 (1.000–1.000)	0.477
CONUT	1.206 (1.125–1.293)	<0.001	1.207 (1.126–1.295)	<0.001	1.087 (0.997–1.185)	0.059
CVD						
PNI	0.965 (0.947–0.983)	<0.001	0.970 (0.951–0.989)	0.002	0.976 (0.954–0.998)	0.036
GNRI	0.976 (0.962–0.989)	<0.001	0.978 (0.964–0.992)	0.002	0.978 (0.963–0.995)	0.009
TCBI	1.000 (1.000–1.000)	0.598	1.000 (1.000–1.000)	0.728	1.000 (1.000–1.000)	0.273
CONUT	1.149 (1.074–1.230)	<0.001	1.124 (1.049–1.205)	<0.001	1.113 (1.029–1.203)	0.007
All-cause mortality						
PNI	0.919 (0.890–0.948)	<0.001	0.921 (0.892–0.952)	<0.001	0.945 (0.897–0.995)	0.032
GNRI	0.942 (0.921–0.963)	<0.001	0.943 (0.922–0.965)	<0.001	0.954 (0.918–0.990)	0.013
TCBI	1.000 (1.000–1.000)	0.775	1.000 (1.000–1.000)	0.951	1.000 (1.000–1.000)	0.664
CONUT	1.323 (1.188–1.473)	<0.001	1.317 (1.176–1.474)	<0.001	1.208 (1.033–1.411)	0.018

Data are presented as HR (hazard ratios), 95% CI (confidence intervals), and *p* value. PNI, Prognostic Nutritional Index; GNRI, Geriatric Nutritional Risk Index; CONUT score, Controlling Nutritional Status score; TCBI, Triglycerides × Total Cholesterol × Body Weight Index; ESKD, end-stage kidney disease; CVD, cardiovascular disease event.

ESKD: Model 1, adjusted none; Model 2, adjusted gender (male) and age; Model 3, adjusted gender (male), age, hypertension, diabetic retinopathy, CKD stage, eGFR, serum creatinine, cystatin C, phosphorus, hemoglobin, iPTH, glomerular lesions, IFTA, interstitial inflammation, arteriolar hyalinosis, arteriosclerosis.

CVD: Model 1, adjusted none; Model 2, adjusted gender (male) and age; Model 3, adjusted gender (male), age, hypertension, coronary disease, diabetic retinopathy, CKD stage, statin use, anti-platelet drug use, eGFR, serum creatinine, cystatin C, hemoglobin, iPTH, IL-8, IFTA, interstitial inflammation, arteriosclerosis.

All-cause mortality: Model 1, adjusted none; Model 2, adjusted gender (male) and age; Model 3, adjusted gender (male), age, eGFR, serum creatinine, cystatin C, calcium, hemoglobin, iPTH, TCH, Lpa, HDL-C, arteriosclerosis.

**Figure 4:**
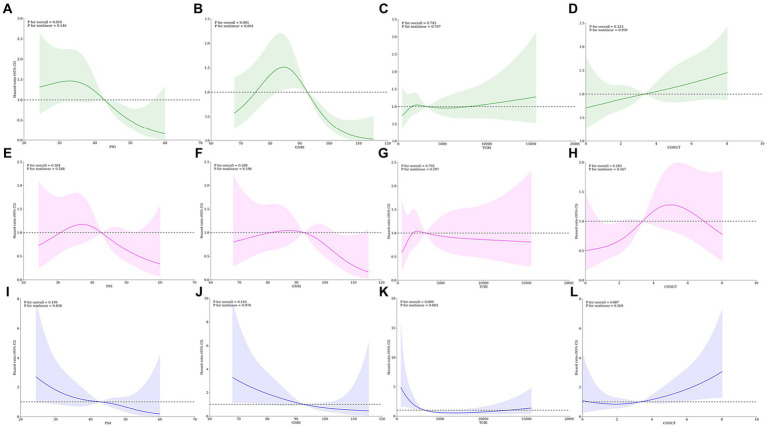
Restricted spline curves for the associations between four nutritional scores and adverse events in DKD patients. Lines represent the HR (hazard ratio), and transparent areas represent the 95% confidence intervals. HR (95% CI) were adjusted for various adverse events in Cox analysis using Model 3. **(A–D)** Associations between PNI, GNRI, TCBI, COUNT score and end-stage renal disease; **(E–H)** Association between PNI, GNRI, TCBI, COUNT score and cardiovascular death; **(I–L)** Association between PNI, GNRI, TCBI, COUNT score and all-cause mortality. PNI, Prognostic Nutritional Index; GNRI, Geriatric Nutritional Risk Index; CONUT score, Controlling Nutritional Status score; TCBI, Triglycerides×Total Cholesterol×Body Weight Index.

Next, we also used restricted cubic splines to model and visualize the relation of predicted nutritional scores (PNI, GNRI, TCBI, and CONUT) with ESKD in DKD patients (Figures 4A–D). For PNI, the risk of ESKD was relatively flat until it reached 34–35 and then started to decrease rapidly afterwards but the P for nonlinearity was non-significant (P for overall = 0.016, P for nonlinear = 0.146). For GNRI, regarding the strong N-shaped relation between predicted GNRI and ESKD, the plot showed an increase of the risk within the lower range of predicted GNRI until around 75 and HR exceeded the horizontal line with HR = 1, which reached the highest risk around 55–56 and then substantially decreased thereafter until it until it reached 92–93 (P for overall<0.001, P for nonlinear<0.001). In addition, the nonlinear relationship between nutrition scores and cardiovascular death was weakened and no apparent correlation was found between the nutrition scores and cardiovascular death (Figures 4E–H). An L-shaped relationship between the HR of all-cause mortality and nutritional scores (PNI, GNRI, and TCBI) was indicated in DKD patients. However, the nonlinear relationship between nutrition scores and all-cause mortality was weakened and no apparent correlation was found between the nutrition scores and all-cause mortality (Figures 4I–L). After adjusting for various adverse events using Model 3 in the Cox analysis, the restricted spline curve indicates a potential linear correlation between the four nutritional scores and the outcomes.

Furthermore, we investigated nutrition scores that exhibit a notable non-linear relationship with outcomes. We employed threshold effect analysis to identify critical inflection point that influence the correlation between variables. Subsequently, we assessed the correlation between the independent and dependent variables both before and after these turning points. The relationship between GNRI and ESKD reveals a critical inflection point at 71.629, indicating a significant threshold effect. When the GNRI falls below 71.629, a positive correlation with ESKD becomes evident (HR = 1.835, 95% CI 1.170–2.878, *p* = 0.008), while exceeding 71.629 leads to a negative association with ESKD (HR = 0.946, 95% CI 0.925–0.968, *p* < 0.001). However, there is no significant threshold effect in TCBI (HR = 1.000, 95% CI 1.000–1.000, *p* = 0.6631) (Table 4).

**Table 4:** Threshold effect analysis of nutritional scores on adverse events.

		Adjusted HR (95% CI)	*p* value
ESKD			
Fitting by standard lincar model	GNRI	0.971 (0.952–0.989)	0.002
Fitting by two-piecewise linear model			
	Inflection point of GNRI	71.629 (69.991–78.478)	
	GNRI<71.629	1.835 (1.170–2.878)	0.008
	GNRI>71.629	0.946 (0.925–0.968)	<0.001
	Log likelihood ratio		<0.001
All-cause mortality			
Fitting by standard lincar model	TCBI	1.000 (1.000–1.000)	0.6631
Fitting by two-piecewise linear model			
	Inflection point of TCBI	4942.720 (3560.630–7792.496)	
	TCBI<4942.720	0.999 (0.999–1.000)	0.002
	TCBI>4942.720	1.000 (1.000–1.000)	0.057
	Log likelihood ratio		<0.001

Data are presented as HR (hazard ratios), 95% CI (confidence intervals), and *p* value. PNI, Prognostic Nutritional Index; GNRI, Geriatric Nutritional Risk Index; CONUT score, Controlling Nutritional Status score; TCBI, Triglycerides × Total Cholesterol × Body Weight Index; ESKD, end-stage kidney disease; CVD, cardiovascular disease event.

Overall, compared with TCBI and CONUT score, GNRI and PNI have a stronger correlation with ESKD, CVD events and all-cause mortality, which is similar to the results of the time-dependent receiver operating characteristic (td-ROC). Therefore, it showed that individual PNI has the best diagnostic accuracy and the strongest correlation with the disease, making it an independent risk factor for ESKD, CVD events, and all-cause death in patients with DKD.

### 3.3 Diagnostic accuracy of four nutritional scores and different combinations with PNI in predicting outcomes

**Figure 5:**
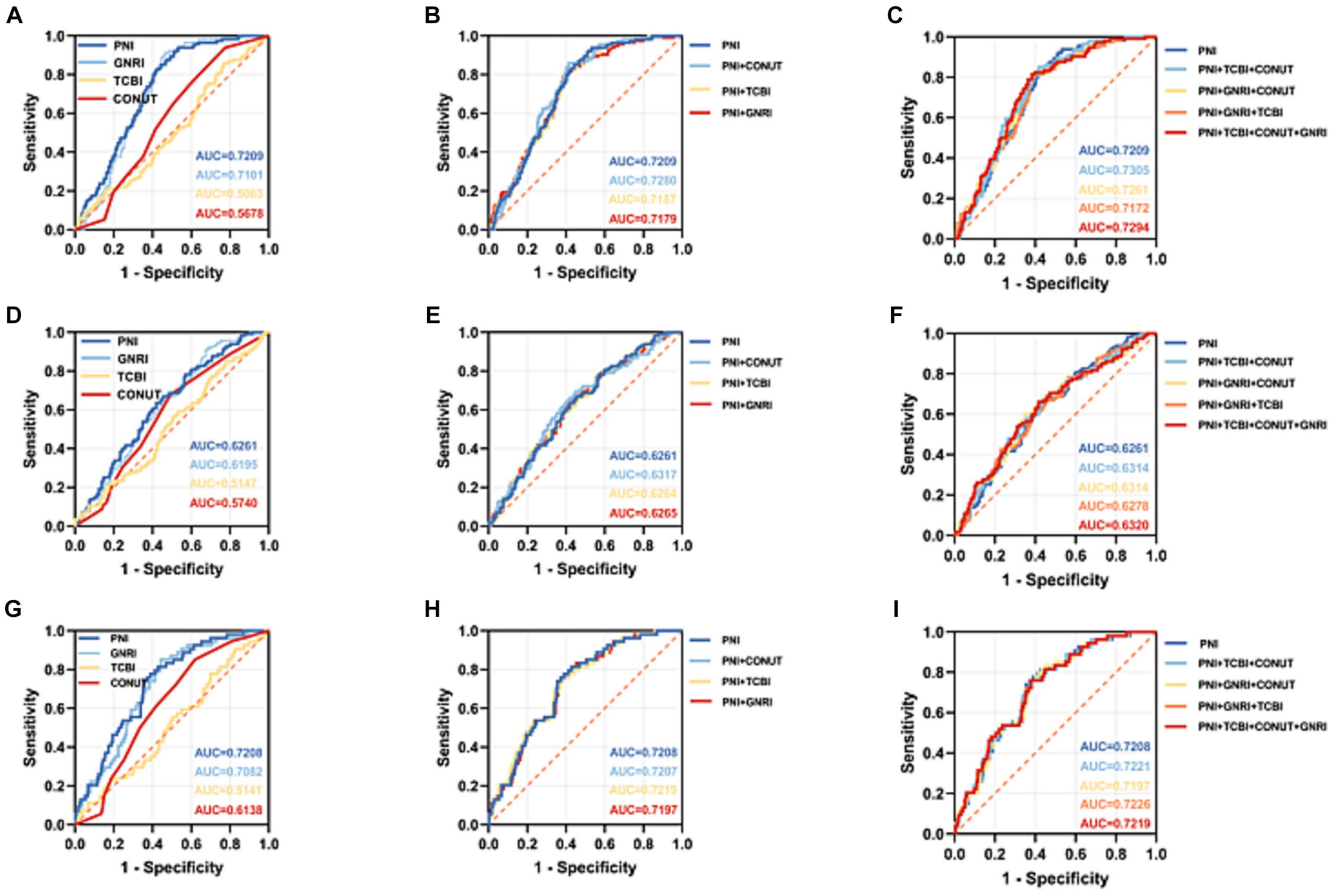
Comparison of the receiver operating characteristic (ROC) curves of four nutritional scores and different combinations with PNI in predicting adverse events in DKD patients. **(A–C)** ROC curves for predicting end-stage renal disease plotted by four nutritional scores and different combinations with PNI in DKD patients. **(D–F)** ROC curves for predicting cardiovascular mortality plotted by four nutritional scores and different combinations with PNI in DKD patients. **(G–I)** ROC curves for predicting all-cause mortality plotted by four nutritional scores and different combinations with PNI in DKD patients. ROC, Receiver operating characteristic; AUC, area under the curve. PNI, Prognostic Nutritional Index; GNRI, Geriatric Nutritional Risk Index; CONUT score, Controlling Nutritional Status score; TCBI, Triglycerides×Total Cholesterol×Body Weight Index.

The prediction of clinical outcomes by the PNI, GNRI, TCBI, CONUT score and different combinations with PNI was evaluated using the time-dependent receiver operating characteristic (td-ROC) of the subjects. Then, the ROC curves were constructed to calculate the area under curve (AUC) (Figures 5A-I). For the prediction of ESKD, the PNI score had slightly higher AUC than GNRI, whereas the TCBI and the CONUT score had similar AUC. Diagnostic accuracy of PNI + TCBI+CONUT (AUC = 0.7305) was slightly higher than that of other combined scores and slightly higher than that of PNI (AUC = 0.7209) alone. Other combinations is not significantly improved or lower than the individual PNI scores (Figures 5B,C). We also obtained similar results in the AUC for cardiovascular death and all-cause mortality. However, the AUC of PNI + TCBI+CONUT+GNRI (AUC = 0.6320) was slightly higher than PNI (AUC = 0.6261) for CVD events (Figure 5F), and the AUC of PNI + GNRI+TCBI (AUC = 0.7226) was slightly higher than PNI (AUC = 0.7208) for all-cause mortality (Figure 5I). Overall, compared with PNI, the diagnostic accuracy of other nutritional scores alone or different combinations with PNI performed worse on ESKD, CVD events and death.

### 3.4 Correlation between four nutritional scores and different renal histologic changes in patients with diabetic kidney disease

**Figure 6:**
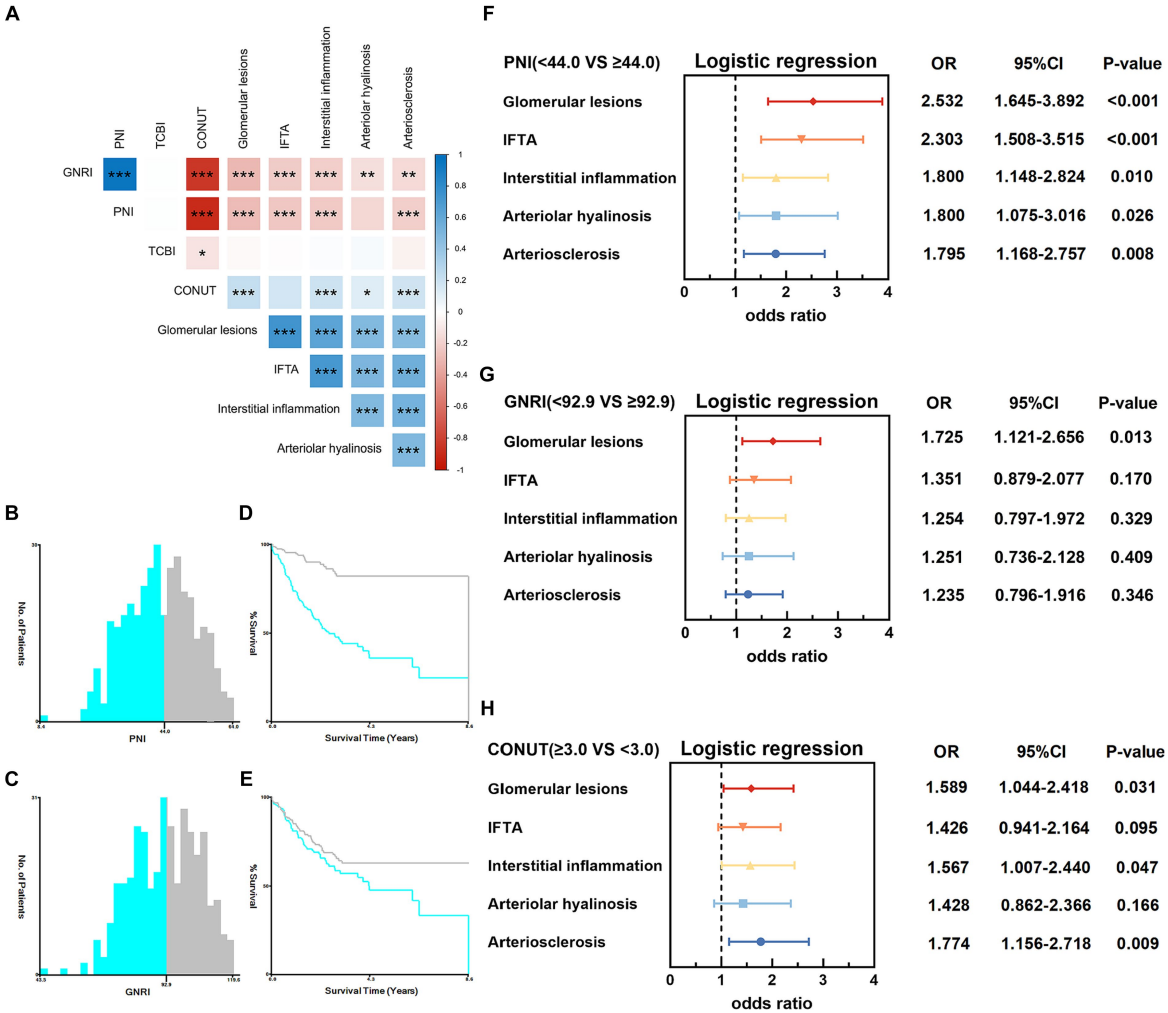
Correlation between four nutritional scores and different renal histologic changes in patients with diabetic kidney disease. **(A)** Correlation heatmap showing the correlation between four nutritional scores and different renal histologic changes. *p < 0.05, **p < 0.01, ***p < 0.001. **(B,C)** Histograms of patient distribution according to the PNI and GNRI. **(D,E)** The Kaplan–Meier curves of the PNI and GNRI to calculate the optimum cutoff value. **(F)** Ordered logistic regression analysis to identify the relationship between PNI (<44.0 vs. ≥44.0) and different renal histological changes. **(G)** Ordered logistic regression analysis to identify the relationship between GNRI (<92.9 vs. ≥92.9) and different renal histological changes. **(H)** Ordered logistic regression analysis to identify the relationship between CONUT (≥3.0 vs. <3.0) score and different renal histological changes. OR, odds ratio; 95%CI, 95% confidence interval.

We detected the correlation analysis between four nutritional scores and different renal histologic changes in patients with DKD, and our results showed PNI was negatively correlated with glomerular lesions (r = −0.290, *p* < 0.001), IFTA (r = −0.234, p < 0.001), interstitial inflammation (r = −0.226, *p* < 0.001), arteriolar hyalinosis (r = −0.168, *p* = 0.001) and arteriosclerosis (r = −0.212, *p* < 0.001). For PNI, we also found similar results. For CONUT score, the positive correlation was found with glomerular lesions (r = 0.224, *p* < 0.001), IFTA (r = 0.176, *p* = 0.001), interstitial inflammation (r = 0.197, *p* < 0.001), arteriolar hyalinosis (r = 0.128, *p* = 0.014) and arteriosclerosis (r = 0.189, *p* < 0.001). No significant correlation between TCBI and renal histologic changes was found in patients with diabetic kidney disease (Figure 6A).

To further explore the underlying correlations between PNI, GNRI and CONUT score and different renal histologic changes in patients with DKD, patients were divided into two groups (the low level group and the high level group) based on X-tile software and median CONUT score. The variable “PNI” was grouped as “<44.0” and “≥44.0” under end-stage renal disease (Figures 6B,D); “GNRI” was categorized into “<92.9” and “≥92.9” under cardiovascular (Figures 6C,E). Patients were divided into two groups based on median CONUT score: the low CONUT score group (CONUT score < 3) and the high CONUT score group (CONUT score ≥ 3). Then, we use ordered logistic regression analysis to investigate the factors affecting different renal histologic changes in patients with DKD.

After adjusting proteinuria in the ordered logistic regression analysis, we found that high PNI level was an adverse factor for obtaining the higher glomerular lesions grade (*p* < 0.001, OR = 2.532, 95%CI 1.645–3.892), IFTA (*p* < 0.001, OR = 2.303, 95%CI 1.508–3.515), interstitial inflammation (*p* = 0.010, OR = 1.800, 95%CI 1.148–2.824), arteriolar hyalinosis (*p* = 0.026, OR = 1.800, 95%CI 1.075–3.016), arteriosclerosis (*p* = 0.008, OR = 1.795, 95%CI 1.168–2.757) (Figure 6F). GNRI was also inversely correlated with glomerular lesions (*p* = 0.013, OR = 1.725, 95%CI 1.121–2.656). There is no significant correlation between GNRI and IFTA, interstitial inflammation, arteriolar hyalinosis and arteriosclerosis (Figure 6G). Besides, a lower CONUT score was linked to improved renal histologic changes in glomerular lesions, interstitial inflammation, and arteriosclerosis grade (Figure 6H). Compared with GNRI and CONUT score, PNI have a stronger correlation with different renal histologic changes and higher PNI may be related to a lower risk of different renal histologic changes in patients with diabetic kidney disease. The PNI remained best incremental values for predicting the order of severity of different renal histologic changes.

## 4 Discussion

In this study, we used four nutritional scores to assess the nutrition status of patients with DKD under different renal histologic changes, analyzed the relationship of nutritional status with ESKD, CVD events, and all-cause mortality in patients with DKD and detected the correlation analysis between four nutritional scores and different renal histologic changes.

The major conclusions are as follows: (1) Malnourished patients were at a higher risk of adverse outcomes. Moreover, GNRI, PNI, and CONUT score had higher predictive value for all-cause mortality than other adverse outcomes. (2) Compared with other nutritional scores, the PNI alone had the highest predictive value in biopsy-confirmed diabetic kidney disease. However, TCBI showed the worst performance on risk assessment and prediction. Moreover, the predictive value of certain combinations with PNI is slightly higher than PNI alone for various adverse outcomes. (3) Malnourished patients were found to have a heightened risk of experiencing significant renal histologic changes. However, no clear correlation could be found between TCBI and renal histologic change. Furthermore, the PNI remained the most accurate predictor of the severity order of various renal histologic changes.

In recent years, it has been widely suggested by numerous studies that CKD patients exhibit abnormal protein-energy metabolism, with significant muscle and fat wasting. In 2008, the International Society of Renal Nutrition and Metabolism (ISRNM) expert group named this condition protein-energy wasting (PEW), which refers to the reduced protein and energy reserves in the body, resulting in a state of malnutrition characterized by decreased protein and fat content (3). Nutrition plays a crucial role in reducing the risk of cardiovascular disease and slowing the decline in kidney function (4). Currently, PEW is highly prevalent among elderly individuals and those with ESKD, and it is closely associated with poor clinical outcomes due to the breakdown of body proteins and reduced energy caused by metabolic inflammation responses (3, 18). In dialysis patients, malnutrition may increase the risk of cardiovascular and all-cause mortality through factors such as chronic inflammation and oxidative stress (19). Malnutrition is a common complication in CKD stages 4–5 and also influence the severity and progression of DKD (20–22). Therefore, the nutritional status is closely related to the progression and prognosis of DKD, and the prevention and treatment of malnutrition in DKD can improve patient outcomes.

Four nutritional scores include two or three of the following elements: serum albumin, lymphocytes count, TC, TG, and body weight. Serum albumin and weight loss are strong independent risk factors for mortality in older persons. Low albumin or weight loss was correlated with increased mortality in older persons (23, 24). Meanwhile, the synthesis of albumin is influenced by chronic inflammation and malnutrition, and lower levels of albumin may be a marker of continuous arterial injury, as well as the progression of atherosclerosis and thrombosis (25). High cholesterol is a common risk factor for CVD. However, low cholesterol is a high risk factor for CVD events in dialysis patients (26). The reason for this paradox may be that the inflammatory or malnutrition state of the organism leads to a disturbance in lipid metabolism, which increases the risk of adverse outcomes. In addition, age related lymphopenia is well described in the literature and an association between lymphopenia and mortality has recently been reported (27). The occurrence of diabetes is accompanied by an increase in reactive oxygen species, which in turn leads to an increase in oxidative stress (28). This oxidative stress (29), along with protein energy consumption (30), are both potential causes of CKD inflammation, and may represent the mechanisms underlying DKD inflammation. Consequently, low serum albumin and low lymphocyte count may contribute to ESKD.

Our study found that a higher risk of adverse outcomes was associated with lower GNRI and PNI, as well as higher CONUT score. GNRI is based on measurements of serum albumin and weight loss, which are strong independent risk factors for mortality in older persons. The utilization of both indicators in the GNRI minimizes confounding variables such as hydration status. Therefore, the GNRI is a reliable prognostic indicator of adverse outcomes in patients with DKD. The CONUT score, a combination of cholesterol, lymphocyte count, and serum albumin, may serve as a reliable indicator for identifying high-risk CVD patients. Early assessment of the CONUT score can provide a preliminary understanding of the nutritional, immune, inflammatory, and lipid metabolism status of patients. Consequently, it can be used as a reference for clinical management.

In addition, we presumed that the PNI may be a better predictor than GNRI and CONUT to predict the ESKD in DKD patients, most likely because the PNI is a more comprehensive marker that reflects nutrition, immune and inflammation (31), all of which are closely related with DKD. Moreover, lymphocyte count proves to be a more consistent measure of body composition over extended periods. In contrast, the markers used in calculating GNRI and TCBI, which include body weight, TC, and TG, are greatly influenced by factors like age, diet, drugs, smoking, drinking, and lifestyle choices. The TCBI score is calculated from variables reflecting lipid metabolism as well as immune function measured from blood tests. We presumed that TCBI may be the worst predictor to predict ESKD in DKD patients, most likely because TC and TG cannot effectively assess the body’s nutritional status, inflammation level, and immune response.

A recommended treatment approach for DKD is the comprehensive management of blood glucose, blood pressure, and blood lipids, aiming to delay DKD progression to ESKD and cardiovascular diseases. High protein intake can further impair kidney function, increasing the risk of DKD progression and cardiovascular events. Carbohydrates, as a readily available source of energy, are one of the main influencing factors of blood glucose. Therefore, many renal experts suggest that DKD patients adopt a low-carbohydrate diet (energy intake of 25–35 kcal/kg/day) and minimize the risk of high protein intake (low protein diet, protein intake of 0.6–0.8 g/kg/day) (22). The new guidelines also differentiate between pre-dialysis diabetes patients and non-diabetes patients, providing specific protein ranges for each group. For clinically stable stage 3–5 CKD patients without diabetes, the new recommendations set a range of 0.55–0.60 g/kg/day or an extremely low protein diet of 0.28–0.43 g/kg/day (32). A lower protein intake reduces readily available energy in the body, thus requiring more carbohydrates to meet energy demands. However, a high carbohydrate intake may worsen blood glucose control in diabetes (33). From an energy perspective, low-carbohydrate and low-protein diets fundamentally contradict each other. Strict dietary restrictions may lower the quality of life in DKD patients and significantly increase the risk of malnutrition. Therefore, it is especially important to comprehensively evaluate the nutritional status of DKD patients and utilize it to restrict protein intake and regulate blood glucose levels. We presumed that the diagnostic accuracy of PNI + TCBI+CONUT+GNRI was slightly higher than PNI alone for cardiovascular death, and the diagnostic accuracy of PNI + GNRI+TCBI was slightly higher than PNI alone for all-cause mortality, most likely because the combinations including more serum nutritional indicators and other factors can comprehensively evaluate the nutritional status of DKD patients.

Recent meta-analysis of kidney biopsies in diabetes patients has shown a wide range of changes in kidney disease (34). Autopsy studies have also indicated that pathological changes in diabetic kidney disease can occur before clinical manifestations like proteinuria and eGFR decline (35–38). Previous research has suggested that some renal histological changes are good predictors of end-stage renal disease, cardiovascular and all-cause mortality (39, 40). Therefore, evaluating the renal histological changes of DKD patients is clinically significant. However, even when clinical manifestations are present, renal biopsies are rarely conducted in routine clinical practice for DKD patients. Interestingly, we did not find any correlation between renal histological changes and all-cause mortality in both the low PNI group and the high PNI group in our study, which may be due to insufficient follow-up time. Moreover, albumin also has anti-inflammatory, antioxidant, and antithrombotic properties. Inflammatory states and conditions that increase capillary permeability can cause low serum albumin concentration, resulting in the expansion of interstitial space and an increase in albumin distribution volume (41). In our study, we found that DKD patients with more severe renal histological changes had a higher risk of adverse outcomes, particularly in low PNI group, where the relationship between renal histological changes (glomerular lesions, IFTA, interstitial inflammation) and end-stage renal disease was more pronounced. We hypothesized that DKD patients with low PNI have lower serum protein concentrations, indicating an increase in the excretion of renal amino acids that activate the RAS (42–44). The activation of the RAS induces glomerulosclerosis and interstitial fibrosis through various mechanisms, ultimately leading to renal histological changes. Therefore, integrating nutritional status and histological changes is crucial, particularly focusing on DKD patients with poor nutritional status (low PNI group), as it may help predict ESKD in these patients.

Despite the crucial findings being mentioned, our study has some limitations: (1) To begin with, our study was conducted at a single center and encompassed a small sample size comprising exclusively of patients with confirmed DKD through renal biopsy. This inclusion criteria might have introduced some degree of selective bias into our findings. We speculated that this was the reason why we found an N-shaped relationship between the nutritional score GNRI and ESKD, rather than an L-shaped relationship. (2) Certain factors that could potentially disrupt the results, including dietary factors and the use of various types of therapeutic medications, were not taken into account. (3) Due to the difficulty of repeated renal biopsies and reassessments, we only evaluated four nutritional scores at the time of patient enrollment, without investigating the impact of changes in renal histology and nutritional assessments overtime on the prognosis of DKD patients. (4) A more comprehensive assessment tool that incorporates additional nutrients, such as blood lipids and glucose, is necessary due to the limited nutritional content of the four nutritional scores.

## 5 Conclusion

In summary, our study demonstrated that the nutritional status of patients with DKD significantly influences their outcomes. We reported an association between end-stage kidney disease, cardiovascular, and all-cause mortality, and four nutritional scores (PNI, GNRI, TCBI, and COUNT). Moreover, our findings indicate that the PNI may provide more accurate predictive values for adverse outcomes and display stronger correlations with various renal histologic changes compared to other nutritional scores.

## Data availability statement

The data analyzed in this study is subject to the following licenses/restrictions: the data presented in this study are available on request from the corresponding author. The data are not publicly available due to the protection of patient’s rights of privacy. Requests to access these datasets should be directed to chenling@cqmu.edu.cn.

## Ethics statement

The studies involving humans were approved by the ethical committee of Xinqiao Hospital (No. 2018-006-02). The studies were conducted in accordance with the local legislation and institutional requirements. The participants provided their written informed consent to participate in this study.

## Author contributions

LX: Writing – review & editing, Writing – original draft, Visualization, Software, Methodology, Investigation, Data curation, Conceptualization. JX: Writing – review & editing, Validation, Supervision, Investigation, Data curation, Conceptualization. QH: Writing – review & editing, Project administration, Methodology, Data curation. WL: Writing – review & editing, Supervision, Investigation. LC: Writing – review & editing, Writing – original draft, Validation, Supervision, Resources, Project administration, Methodology, Investigation, Funding acquisition, Data curation, Conceptualization.
