# Supplementary material for: Comparative analysis of four nutritional scores in predicting adverse outcomes in biopsy-confirmed diabetic kidney Disease
Source: Front Nutr. 2024 Mar 20;11:1352030. doi: 10.3389/fnut.2024.1352030 (PMC10987755; doi:10.3389/fnut.2024.1352030)
Supplement: Supplementary file 1 [file Table_1.DOCX]

Supplementary Material

# Supplementary **Table**

**Supplementary Table 1** Parameters for assessment of the CONUT Score.

| Table S1. Evaluation of CONUT score | | | | |
| --- | --- | --- | --- | --- |
| Parameter | Score |  |  |  |
| Serum albumin (g/L) | ≥35 | 30-34 | 25-29 | <25 |
| Albumin score | 0 | 2 | 4 | 6 |
| Total cholesterol (mg/dl) | ≥180 | 140-179 | 100-139 | <100 |
| Cholesterol score | 0 | 1 | 2 | 3 |
| Total lymphocytes (10^9/L) | ≥1.600 | 1.200-1.599 | 0.800-1.199 | <0.800 |
| Lymphocyte score | 0 | 1 | 2 | 3 |
